# Bacterial resistance to ciprofloxacin in Greece: results from the National Electronic Surveillance System. Greek Network for the Surveillance of Antimicrobial Resistance.

**DOI:** 10.3201/eid0503.990325

**Published:** 1999

**Authors:** A C Vatopoulos, V Kalapothaki, N J Legakis

**Affiliations:** Athens University, Athens (Goudi), Greece. avatopou@cc.uoa.gr

## Abstract

According to 1997 susceptibility data from the National Electronic System for the Surveillance of Antimicrobial Resistance, Greece has high rates of ciprofloxacin resistance. For most species, the frequency of ciprofloxacin-resistant isolates (from highest to lowest, by patient setting) was as follows: intensive care unit > surgical > medical > outpatient. Most ciprofloxacin-resistant strains were multidrug resistant.


### Bacterial Resistance to Ciprofloxacin in Greece: Results from the National Electronic Surveillance System

**A.C. Vatopoulos, V. Kalapothaki, Greek Network for the Surveillance of Antimicrobial Resistance,[1] and N.J. Legakis**
Athens University, Athens (Goudi), Greece

According to 1997 susceptibility data from the National Electronic System for the Surveillance of Antimicrobial Resistance, Greece has high rates of ciprofloxacin resistance. For most species, the frequency of ciprofloxacin-resistant isolates (from highest to lowest, by patient setting) was as follows: intensive care unit > surgical > medical > outpatient. Most ciprofloxacin-resistant strains were multidrug resistant.

Soon after the broad-spectrum, highly effective antibiotics fluoroquinolones were introduced, their extensive use and misuse in hospitals and communities, as well as in veterinary medicine, have led to the emergence and spread of resistant strains (1,2). Highly divergent rates of fluoroquinolone resistance in both community-acquired and nosocomial pathogens have been reported worldwide (2). Many factors, including patient characteristics, local epidemiologic factors, antibiotic policies, over-the-counter use (which often leads to inadequate use), lower standard of living in developing countries, lack of information on the prudent use of antibiotics, and use of antibiotics in animal husbandry may contribute to the emergence of quinolone-resistant organisms.

Surveillance is an integral part of controlling resistance, and local and national surveys to identify, monitor, and study the epidemiology of the emergence and spread of resistant isolates are needed (3). To identify national trends and local differences in the epidemiology of quinolone resistance in Greece, we report 1997 ciprofloxacin susceptibility data from the National Electronic System for the Surveillance of Antimicrobial Resistance.

The National Electronic System for the Surveillance of Antimicrobial Resistance was introduced in Greece 3 years ago. Involving 17 hospitals throughout Greece, the system analyzes the routine results of the antibiotic sensitivity tests performed in hospital microbiology laboratories by using WHONET software (4).

In our analysis we included 11,097 isolates (4,204 from medical wards, 2,897 from surgical wards, 1,724 from intensive care units [ICU], and 2,272 from outpatient departments) (Table 1). We focused on the bacteria most frequently encountered in Greek hospitals (National Electronic System for the Surveillance of Antimicrobial Resistance [www.mednet.gr/whonet]; N.J. Legakis, Enare Sentry, unpub. data): *Escherichia coli, Klebsiella pneumoniae, Enterobacter* species, *Pseudomonas aeruginosa*,

Table 1. Isolates included in the analysis[a]

| Species | Type of ward: Medical | Surgical | ICU[b] | Outpatients | All |
|---|---|---|---|---|---|
| *Escherichia coli* | 2,100 | 1,114 | 94 | 1,571 | 4,879 |
| *Pseudomonas aeruginosa* | 672 | 527 | 570 | 195 | 1,964 |
| *Staphylococcus aureus* | 452 | 467 | 318 | 248 | 1,485 |
| *Enterobacter* spp. | 396 | 332 | 198 | 142 | 1,068 |
| *Klebsiella pneumoniae* | 419 | 224 | 177 | 96 | 916 |
| *Acinetobacter* spp. | 165 | 233 | 367 | 20 | 785 |
| All | 4,204 | 2,897 | 1,724 | 2,272 | 11,097 |

[a]One isolate per species per patient (the first isolated) is shown. [b]ICU, intensive care unit.

Address for correspondence: A.C. Vatopoulos, Department of Hygiene & Epidemiology, Medical School, Athens University, 115 27 Athens (Goudi), Greece; fax: 30-1-7704225; e-mail: avatopou@cc.uoa.gr.

[1]G. Antoniadis, E. Arhondidou, S. Chatzipanagiotou, E. Chinou, A. Chrysaki, V. Daniilidis, G. Genimata, H. Gessouli, P. Golemati, E. Kaili-Papadopoulou, A. Kansuzidou, D. Kailis, E. Kaitsa, M. Kanelopoulou, Sp. Kitsou-Kyriakopoulou, Z. Komninou, E. Kouskouni, Chr. Koutsia-Karouzou, S. Ktenidou-Kartali, V. Liakou, H. Malamou-Lada, H. Mercuri, C. Nicolopoulou, A. Pagkali, E. Panagiotou, E. Papafragas, A. Perogamvros, C. Poulopoulou, D. Sofianou, G. Theodoropoulou-Rodiou, S. Thermogianni, E. Trikka-Graphakos, O. Vavatsi-Manou, M. Ventouri, E. Vogiatzakis, A. Xanthaki, Chr. Zagora, E. Chatzidaki, G. Papoutsakis.

*Acinetobacter baumanii*, and *Staphylococcus aureus*. These species are also the most important nosocomial pathogens in most parts of the world in terms of rate of isolation, pathogenicity, and virulence (5,6).

Isolation and identification were performed by standard methods at the microbiology laboratories of each hospital participating in the network. The susceptibility testing methods were Kirby-Bauer disk diffusion (7 hospitals); Sensititre (Sensititre, Salem, NH) (1); Pasco (Difco, Detroit, MI) (8); and VITEK (Bieux-Merieux Marcy l'Etoile, France) (1). The actual zone diameters or MICs (not the interpretations of the tests) were entered into WHONET. The chi-square test was used to evaluate differences in resistance rates between types of wards, as well as between clinical specimens. Pearson's correlation coefficients were calculated for possible associations between resistance rates and hospital size.

The resistance rate to ciprofloxacin by type of ward, clinical specimen, and bacterial species is shown in Table 2. There is a stepwise decrease in the frequency of isolation of ciprofloxacin-resistant isolates (ciprofloxacin resistance in

Table 2. Ciprofloxacin resistance by specimen and type of ward[a]

| | Outpatients | | Medical | | Surgical | | ICU | |
|---|---|---|---|---|---|---|---|---|
| | No. | %R[b] | No. | %R | No. | %R | No. | %R |
| *Escherichia coli* | | | | | | | | |
| Urine | 1,191 | 5.0 | 1,572 | 5.5 | 597 | 8.5 | 39 | 10.2 |
| Blood | - | | 195 | 6.9 | 14 | 18.1 | 5 | 0.0 |
| Respiratory | - | | 56 | 2.1 | - | | 23 | 9.0 |
| Pus | - | | 33 | 12.1 | 203 | 8.4 | 11 | 27.8 |
| Other | 380 | 4.5 | 244 | 7.5 | 300 | 6.5 | 16 | 20.0 |
| All | 1,571 | 3.7 | 2,100 | 5.6 | 1,114 | 8.2 | 94 | 13.3 |
| *Salmonella* spp. | | | | | | | | |
| Stool | 195 | 0.7 | | | | | | |
| *Klebsiella pneumoniae* | | | | | | | | |
| Urine | 62 | 6.6 | 254 | 15.5 | 85 | 19.8 | 28 | 64.0 |
| Blood | - | | 45 | 11.3 | 10 | 9.8 | 18 | 72.3 |
| Respiratory | - | | 62 | 9.8 | 12 | 50.0 | 90 | 69.8 |
| Pus | - | | 14 | 50.0 | 42 | 19.0 | 0 | 0.0 |
| Other | 34 | 3.1 | 44 | 18.5 | 79 | 28.3 | 41 | 65.4 |
| All | 96 | 5.4 | 419 | 15.8 | 226 | 23.9 | 177 | 67.7 |
| *Serratia marcences* | | | | | | | | |
| All | | | 76[c] | 7.7[c] | | | 20 | 45.2 |
| *Enterobacter* spp. | | | | | | | | |
| Urine | 76 | 12.0 | 190 | 29.7 | 85 | 32.0 | 24 | 75.4 |
| Blood | - | | 37 | 21.8 | 13 | 54.2 | 24 | 66.6 |
| Respiratory | - | | 76 | 6.3 | 10 | 40.2 | 58 | 48.6 |
| Pus | - | | 22 | 36.8 | 138 | 18.5 | 27 | 67.6 |
| Other | 66 | 10.8 | 71 | 16.9 | 86 | 23.3 | 65 | 69.0 |
| All | 142 | 11.6 | 396 | 22.2 | 332 | 24.8 | 198 | 62.2 |
| *Pseudomonas aeruginosa* | | | | | | | | |
| Urine | 51 | 31.0 | 270 | 44.0 | 171 | 40.7 | 70 | 79.3 |
| Blood | 0 | 0.0 | 24 | 20.6 | 13 | 46.5 | 29 | 75.6 |
| Respiratory | 11 | 18.2 | 258 | 34.4 | 29 | 44.6 | 379 | 62.9 |
| Pus | 18 | 11.3 | 35 | 31.6 | 147 | 22.6 | 16 | 69.5 |
| Ear | 72 | 1.7 | 7 | 47.3 | 30 | 3.7 | 0 | 0.0 |
| Other | 43 | 18.8 | 78 | 26.9 | 137 | 25.9 | 76 | 66.9 |
| All | 195 | 16.7 | 672 | 37.5 | 527 | 28.2 | 570 | 66.4 |
| *Acinetobacter* spp. | | | | | | | | |
| Urine | - | | 72 | 62.6 | 32 | 65.9 | 34 | 94.4 |
| Blood | - | | 18 | 38.7 | 16 | 69.0 | 40 | 92.3 |
| Respiratory | - | | 38 | 49.7 | 11 | 100.0 | 190 | 91.0 |
| Pus | - | | 13 | 61.8 | 87 | 60.1 | 19 | 94.8 |
| Other | - | | 24 | 62.5 | 87 | 69.1 | 84 | 78.9 |
| All | 20 | 45.1 | 165 | 56.8 | 233 | 66.6 | 367 | 88.4 |
| *Staphylococcus aureus* | | | | | | | | |
| Urine | - | | 37 | 32.9 | 16 | 31.0 | - | |
| Blood | - | | 101 | 51.0 | 15 | 67.0 | 40 | 62.7 |
| Respiratory | - | | 123 | 45.3 | 28 | 57.1 | 221 | 65.8 |
| Pus | 104 | 18.2 | 88 | 21.6 | 272 | 30.8 | 14 | 71.4 |
| Ear | 52 | 3.8 | - | | - | | - | |
| Other | 92 | 10.3 | 103 | 25.6 | 136 | 31.4 | 43 | 67.4 |
| All | 248 | 12.8 | 452 | 30.5 | 467 | 33.0 | 318 | 63.6 |
| MRSA[d] | 40 | 56.7 | 140 | 69.1 | 176 | 75.3 | 375 | 94.3 |
| MSSA[e] | 184 | 1.7 | 256 | 12.4 | 219 | 6.5 | 92 | 4.6 |

[a]One isolate per patient (the first isolated) is shown. [b]R, resistant. [c]Medical and surgical wards combined. [d]MRSA, methicillin-resistant *S. aureus*. [e]MSSA, methicillin-sensitive *S. aureus*.

isolates from ICU patients > isolates from surgical patients > isolates from medical patients > isolates from outpatients). These differences were significant ($p < 0.01$), with the exception of decreases in resistance rates for *E. coli* between surgical wards and ICUs; for *Enterobacter* spp. between medical and surgical wards; for *Acinetobacter* spp. between outpatients, medical, and surgical wards; and for *S. aureus* between medical and surgical wards. Moreover, for *P. aeruginosa,* the resistance rates were significantly higher in medical than in surgical wards ($p = 0.00097$).

As for clinical specimens, each bacterial species followed a different pattern (Table 2). In medical wards, enterobacterial strains isolated from purulent infections were more often resistant to ciprofloxacin, but this difference was statistically significant only for *K. pneumoniae* ($p = 0.012$). In surgical wards, blood and respiratory isolates were more often resistant, but this difference was significant only for *Enterobacter* spp. ($p = 0.02$). On the other hand, ciprofloxacin-resistant *P. aeruginosa* strains were more frequently isolated ($p = 0.0021$) in medical wards from urine and in surgical wards from urine and blood as opposed to all other specimens ($p = 0.0005$). No significant differences were observed in the rate of isolation of ciprofloxacin-resistant *A. baumanii* strains among the various clinical specimens. *S. aureus* strains resistant to ciprofloxacin were mostly methicillin-resistant (MRSA) (Table 2). Very low resistance rates were observed in *P. aeruginosa* isolated from ear infections, especially from outpatients.

Approximately 75% of *K. pneumoniae*, 87% of *Enterobacter* spp., 55% of *P. aeruginosa*, 76% of *A. baumanii*, and 75% of MRSA strains were drug resistant to at least three different classes (Table 3). However, 15% of the ciprofloxacin-resistant *E. coli* were resistant only to this antibiotic, and 25% had additional resistance only to cotrimoxazole. Moreover, 48% of ciprofloxacin-resistant but methicillin-sensitive *S. aureus* were resistant only to chloramphenicol.

When we plotted resistance rates to ciprofloxacin against the number of beds in each hospital, we found no correlation (Figure). The rate of isolation of ciprofloxacin-resistant isolates varied greatly by hospital for all species examined: from 1% to 15% for *E. coli*, 1% to 23% for *K. pneumoniae*, 1% to 33% for *Enterobacter* spp., 11% to 33% for *P. aeruginosa*, 29% to 73% for

Table 3. Resistant phenotypes of ciprofloxacin-resistant isolates to other classes of antibiotics[a]

*Klebsiella pneumoniae*

| Phenotype[b] | No. | % |
|---|---|---|
| F | 4 | 3.7 |
| DBXF | 9 | 8.4 |
| IDB F | 16 | 15.0 |
| IDBXF | 64 | 59.8 |
| all other | 14 | 13.1 |
| All | 107 | 100.0 |

*Enterobacter* spp

| Phenotype | No. | % |
|---|---|---|
| F | 0 | 0 |
| IF | 4 | 2.5 |
| IDB F | 7 | 4.4 |
| IDBXF | 131 | 82.9 |
| all other | 16 | 10.1 |
| All | 158 | 100.0 |

*Escherichia coli*

| Phenotype | No. | % |
|---|---|---|
| F | 25 | 15.1 |
| IDBXF | 16 | 9.6 |
| IXF | 29 | 17.5 |
| XF | 42 | 25.3 |
| all other | 54 | 32.5 |
| All | 166 | 100.0 |

*Pseudomonas aeruginosa*

| Phenotype | No. | % |
|---|---|---|
| F | 10 | 7.3 |
| 1DM F | 14 | 10.2 |
| 1DMNF | 23 | 16.8 |
| 1 M F | 40 | 29.2 |
| all other | 50 | 36.5 |
| All | 137 | 100.0 |

*Acinetobacter baumanii*

| Phenotype | No. | % |
|---|---|---|
| F | 0 | 0.0 |
| SMD XF | 5 | 10.0 |
| D XF | 15 | 30.0 |
| MD XF | 23 | 46.0 |
| all other | 7 | 14.0 |
| All | 50 | 100.0 |

*Staphylococcus aureus*

MRSA

| Phenotype | No. | % |
|---|---|---|
| F | 0 | 0.0 |
| OG E F | 23 | 11.3 |
| OG ECF | 44 | 21.7 |
| OGXECF | 84 | 41.4 |
| all other | 52 | 25.6 |
| All | 203 | 100.0 |

MSSA

| Phenotype | No. | % |
|---|---|---|
| F | 7 | 10.3 |
| E F | 9 | 13.2 |
| CF | 33 | 48.5 |
| all other | 19 | 27.9 |
| All | 68 | 100.0 |

[a]All wards, intensive care units isolates are not included. [b]1, piperacillin; B, tobramycin; C, chloramphenicol; D, ceftazidime; E, erythromycin; F, ciprofloxacin; G, gentamicin; I, cefoxitin; M, amikacin; N, imipenem; O, oxacillin; S, amoxicillin/sulbactam; X, cotrimoxazole; MRSA, methicillin-resistant *S. aureus;* MSSA, methicillin-sensitive *S. aureus*.

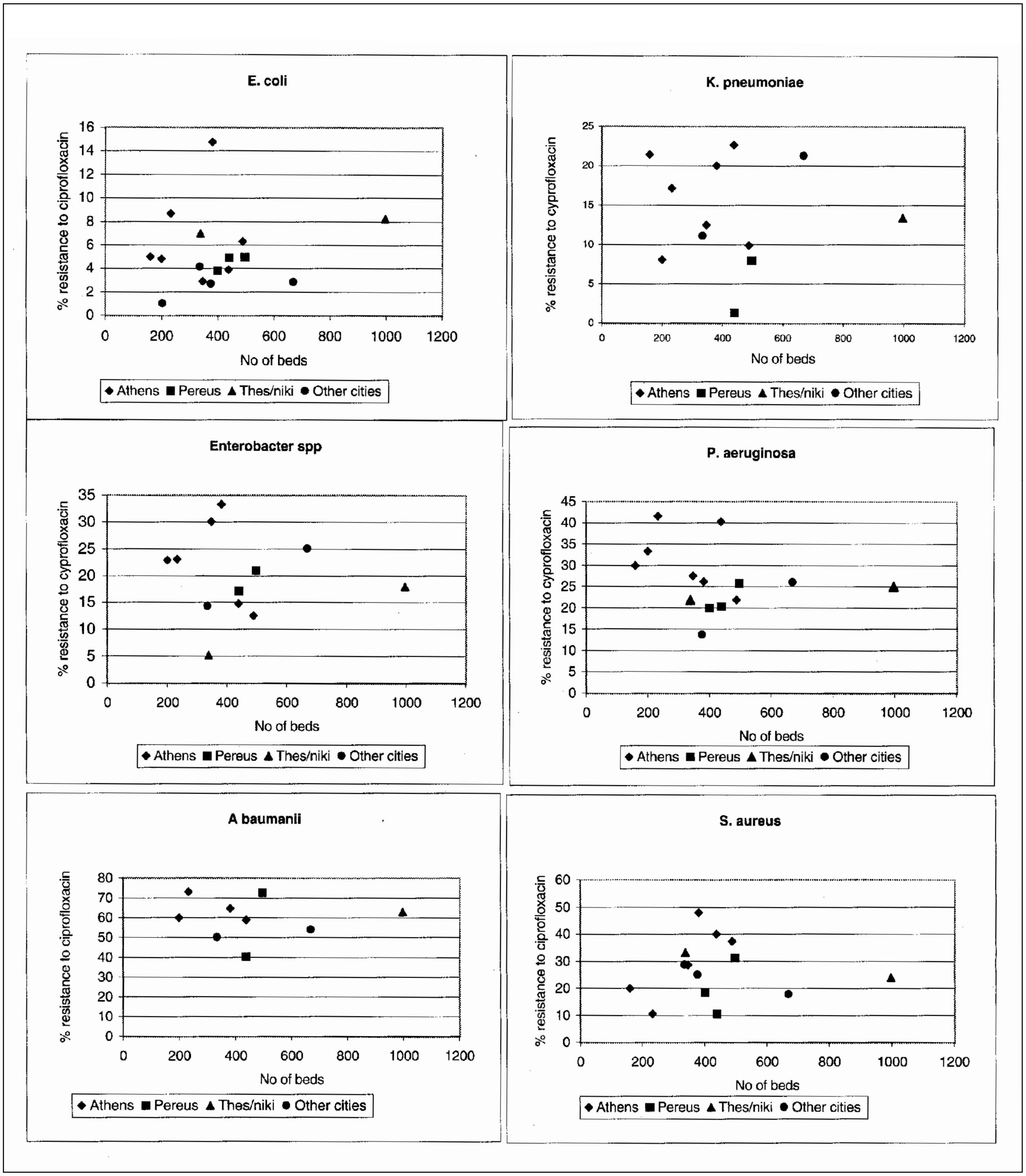

Figure. Resistance rates to ciprofloxavin in each hospital by number of beds and geographic area of the hospital. Only hospitals with more than 20 isolates are included. (Isolates from all wards but not intensive care units.)

*A. baumanii*, and 11% to 48% for *S. aureus*. Ciprofloxacin resistance was observed in hospitals throughout Greece.

In Europe and North America, a striking difference in the incidence of bacterial resistance to quinolones has been observed between nosocomial and community-acquired infections; resistance is only rarely encountered among the latter (2,7). The incidence of resistance to fluoroquinolones in bacteria isolated from hospital-acquired infections varies among bacterial species, clinical settings, and countries and may be related to local epidemic spread of a few clones (2). The highest incidence of resistance is among *P. aeruginosa, Acinetobacter* spp., *Serratia marcescens,* and particularly MRSA strains (8). Our results place Greece among the countries with high resistance levels to quinolones. Although quinolones are among the antibiotics restricted by the Greek Ministry of Health and Welfare, the mean national level of quinolone resistance has increased in most bacterial species during the last 5 years (9).

The 3.7% quinolone resistance rate among *E. coli* isolated from outpatients is almost double that in other industrialized countries (2). This high rate may be due to the use of quinolones, and especially norfloxacin, as a first-line antibiotic in Greece to treat uncomplicated urinary tract infections in the outpatient setting. Free access to fluoroquinolones has also been incriminated in increased quinolone resistance in industrialized and developing countries (10,11). The low rate of quinolone resistance in salmonellas, compared with other countries (12,13), may be due to infrequent use of quinolones in farm animals in Greece. Among *Enterobacteriaceae,* quinolone resistance seems to be higher in *K. pneumoniae* and *Enterobacter* spp. than in *S. marcescens*.

The high level of resistance in ICUs was expected since ICUs are well-known focuses of antimicrobial resistance (14). Hospitalization in ICUs was an independent risk factor for acquiring infection by multidrug-resistant strains in Greece (15). Moreover, ICU patients are often colonized with endemic, multidrug-resistant strains, which often spread to other wards (16).

We found higher rates of isolation of quinolone-resistant strains of some species in the surgical wards than in medical wards. Patients at high risk for a resistant nosocomial infection (e.g., cancer patients, immunosupressed patients) are usually in medical wards. High resistance in the surgical wards could be the result of nursing practices or unnecessary prophylactic administration of antibiotics, both of which should be further evaluated.

Most quinolone-resistant strains in Greece are also resistant to other clinically relevant antibiotics. The possible clinical and epidemiologic importance of the newly described multidrug efflux pumps in multidrug resistance, mainly in *P. aeruginosa,* is under investigation worldwide (17). Moreover, the marginal susceptibility of *S. aureus* to quinolones and the ease with which mutations affecting susceptibility can occur in this species contribute to the observed high rates of quinolone resistance. MRSA strains are no more likely to develop resistance to quinolones than other staphylococci (8). In any case, the favorable accumulation of different traits in quinolone-resistant strains or, alternatively, the favorable potential for mutation to quinolone resistance in multidrug-resistant strains has not been proved. Epidemiologic parameters, and more specifically the sequential introduction of various antibiotic classes in most of the world and in Greek hospitals, could explain multidrug resistance. The extensive aminoglycoside and beta-lactamase use in the 1980s is responsible for the high prevalence of multidrug-resistant plasmids and transposons found in the nosocomial strains of various bacterial genera in Greek hospitals (18-20). The strains harboring these plasmids can survive in the hospital environment and become the best candidates for selection of resistant mutants under the pressure of quinolones.

That quinolone-resistant strains are found in hospitals in all parts of Greece and resistance is not associated with the size of the hospital or its geographic area are consistent with the high prescription rate for quinolones. However, the isolation rate of resistant strains varied considerably by hospital, perhaps because of local epidemiologic factors (e.g., prescribing or nursing habits) or possible (epidemic) spread of strains among patients.

This study has limitations. First, it is based on routine data generated in the microbiology laboratories of participating hospitals. Sometimes different antibiotics are tested in each hospital, which limits the possibility for interhospital comparisons. Moreover, different methods for susceptibility testing are used in each hospital. Data such as antibiotic consumption or days of hospitalization are not available since they are not included as information in the WHONET software and they are difficult and time-consuming to collect routinely.

Quinolone use is a well-proven independent risk factor for resistance (21,22). Nevertheless, local differences indicate that other epidemiologic parameters should be further evaluated.

The National Electronic System for the Surveillance of Antimicrobial Resistance has been supported in part by a grant from the Greek Ministry of Health and Welfare.

The following hospitals participate in the system: Polycliniki General Hospital, Agia Olga General Hospital, Elpis General Hospital, First IKA Hospital of Athens, Agios Savas Cancer Hospital, Sismanoglion General Hospital, Hippocration General Hospital, Areteion University Hospital, Venizelio General Hospital, University Hospital of Alexandroupolis, University Hospital of Ioannina, General Hospital of Xanthi, Threassio General Hospital, Tzannio General Hospital, Asclepeion Voulas General Hospital, Theagenio Cancer Hospital, and Hippocration Hospital Thessaloniki.

Dr. Vatopoulos is a medical microbiologist and assistant professor in the Department of Hygiene and Epidemiology, Medical School, Athens University. His chief research interest is the molecular epidemiology of antibiotic resistance in bacteria (mainly gram-negative). He is now involved in the establishment and operation of an electronic network for the surveillance of antibiotic resistance in Greece.

### References

1. Blondeau JM, Yaschuk Y, Canadian ciprofloxacin susceptibility study. Comparative study from 15 medical centers. Antimicrob Agents Chemother 1996;40:1729-32.
2. Acar JF, Goldstein FW. Trends in bacterial resistance to fluoroquinolones. Clin Infect Dis 1997;24:S67-73.
3. Report of the American Society for Microbiology Task Force on Antibiotic Resistance. Washington: American Society for Microbiology; 1995. p. 1-23.
4. Stelling JM, O'Brien TF. Surveillance of antimicrobial resistance: the WHONET program. Clin Infect Dis 1997;24:S157-68.
5. Emori TG, Gaynes RP. An overview of nosocomial infections, including the role of the microbiology laboratory. Clin Microbiol Rev 1993;6:428-42.
6. Wartz MN. Hospital-acquired infections: diseases with increasingly limited therapies. Proc Natl Acad Sci U S A 1994;91:2420-7.
7. Goldstein FW, Acar JF. Epidemiology of quinolone resistance: Europe and North and South America. Drugs 1995;49:S36-42.
8. Sanders CC, Sanders WE Jr, Thomson. Fluoroquinolone resistance in *Staphylococci*: new challenges. Eur J Clin Microbiol Infect Dis 1995;Suppl 1:6-11.
9. Legakis NJ, Tzouvelekis LS, Tsakris A, Legakis JN, Vatopoulos AC. On the incidence of antibiotic resistance among aerobic gram-negative rods isolated in Greek hospitals. J Hosp Infect 1993;24:233-7.
10. Kresken M, Hafner D, Mittermayer H, Verbist L, Bergogne-Berezin E, Giamarellou H, et al. Prevalence of fluoroquinolone resistance in Europe. Study Group 'Bacterial Resistance' of the Paul-Ehrlich-Society for Chemotherapy. Infection 1994;22:S90-8.
11. Casellas JM, Blanco MG, Pinto ME. The sleeping giant: antimicrobial resistance. Infect Dis Clin North Am 1994;8:29-45.
12. Tassios PT, Markogiannakis A, Vatopoulos AC, Katsanikou E, Velonakis EN, Kourea-Kremastinou J, et al. Molecular epidemiology of antibiotic resistance of *Salmonella enteritidis* during a seven year period in Greece. J Clin Microbiol 1997;35:1316-21.
13. Tassios PT, Vatopoulos AC, Mainas E, Gennimata D, Papadakis J, Tsiftsoglou A, et al. Molecular analysis of ampicillin-resistant sporadic *Salmonella typhi* and *Salmonella paratyphi* B clinical isolates. Clinical Microbiology and Infection 1997;3:317-23.
14. Archibald L, Phillips L, Monnet D, McGrowan JE, Tenover F, Gaynes R. Antimicrobial resistance in isolates from inpatients and outpatients in the United States: increasing importance of the intensive care unit. Clin Infect Dis 1997;24:211-5.
15. Vatopoulos AC, Kalapothaki V, Legakis NJ, the Hellenic Antibiotic Resistance Study Group. Risk factors for nosocomial infections caused by gram-negative bacilli. J Hosp Infect 1996;34:11-22.
16. Tassios PT, Gennimata V, Spaliara-Kalogeropoulou L, Kairis D, Koutsia C, Vatopoulos A, et al. Multiresistant *Pseudomonas aeruginosa* serogroup O:11 outbreak in an intensive care unit. Clinical Microbiology and Infection 1997;3:621-8.
17. Nikaido H. Antibiotic resistance caused by gram-negative multidrug efflux pumps. Clin Infect Dis 1998;Suppl 1:S32-41.
18. Vatopoulos A, Phillipon A, Tsouvelekis L, Komninou Z, Legakis NJ. Prevalence of a transferable SHV-5 type ß-lactamase in clinical isolates of *Klebsiella pneumoniae* and *Escherichia coli* in Greece. J Antimicrob Chemother 1990;26:635-48.
19. Tsakris A, Johnson AP, George RC, Mehtar S, Vatopoulos AC. Distribution and transferability of plasmids encoding trimethoprim resistance in urinary pathogens from Greece. J Med Microbiol 1991;34:153-7.
20. Vatopoulos AC, Tsakris A, Tzouvelekis LS, Legakis NJ, Pitt TL, Miller GH, et al. Diversity of aminoglycoside resistance in *Enterobacter cloacae* in Greece. Eur J Clin Microbiol Infect Dis 1992;11:131-8.
21. Richard P, Delangle MH, Merrien D, Barille S, Reynaud A, Minozzi C, et al. Fluoroquinolone use and fluoroquinolone resistance: Is there an association? Clin Infect Dis 1994;19:54-9.
22. Carratala J, Fernandez-Sevilla A, Tubau F, Callis M, Gudiol F. Emergence of quinolone-resistant *Escherichia coli* bacteremia in neutropenic patients with cancer who have received prophylactic norfloxacin. Clin Infect Dis 1995;20:557-60.

